# Optimized Endospore Production and Carrier Formulations of Endophytic *Bacillus* Species to Enhance Field Control of Wheat Blast

**DOI:** 10.3390/jof12100766

**Published:** 2026-10-12

**Authors:** Sharmin Islam, Md Omar Kayees, Dipali Rani Gupta, Lamya Muazzeda Medha, Julfikar Ali, Md. Shahrear Parvaj Sujon, Tofazzal Islam

**Affiliations:** Institute of Biotechnology and Genetic Engineering (IBGE), Gazipur Agricultural University (GAU), Gazipur 1706, Bangladesh; sharminislam39983@gmail.com (S.I.); kbdkayess@gmail.com (M.O.K.); drgupta80@gau.edu.bd (D.R.G.); lamyamedha98@gmail.com (L.M.M.); julfikarhstu.ac.bd@gmail.com (J.A.); shahrearsujon41@gmail.com (M.S.P.S.)

**Keywords:** wheat blast, *Bacillus* spp., bioformulations, endospore, biocontrol

## Abstract

Harnessing endophytic bacteria for plant disease management offers a promising sustainable strategy. We previously demonstrated that endophytic *Bacillus* spp. significantly suppress wheat blast disease under field conditions. Despite their promising biocontrol potential, the successful commercialization of microbial biopesticides depends on economical mass production, high microbial viability, prolonged shelf-life, and consistent field performance. This study optimized fermentation conditions, endospore production and carrier formulations of three endophytic *Bacillus* strains (*B. subtilis* BTS-3 and *B. velezensis* BTS-4 and BTLK6A) for the sustainable management of wheat blast caused by *Magnaporthe oryzae* pathotype *Triticum* (MoT). Sugarcane molasses was evaluated as a low-cost alternative to commercial nutrient broth, while supplementation with 500 ppm MnCl_2_·4H_2_O enhanced growth and endospore production for *Bacillus* spp. Talc- and molasses-based liquid formulations were subsequently developed and evaluated for storage stability. A talc formulation containing 1% (*w*/*w*) carboxymethyl cellulose and 1.5% (*w*/*w*) calcium carbonate (CaCO_3_) provided the highest survival up to 180 days, maintaining a population density of 1 × 10^5^–10^6^ CFU g^−1^, whereas liquid formulations exhibited a faster decline in cell viability. Despite these differences, both formulation types significantly reduced blast disease severity by 48.78–75.98% compared to the only MoT- inoculated control in the field. Seed priming consistently outperformed foliar application and enhanced tiller production (9.40–16.62%), thousand-grain weight (13.47–32.41%), and grain yield (up to 321 g/m^2^), achieving up to 38.58% higher yield than the MoT-inoculated control. Taken together, these findings establish a cost-effective approach for developing stable talc- and molasses-based formulations, thereby enhancing the commercial viability and large-scale deployment of biological control strategies for sustainable wheat blast management.

## 1. Introduction

Wheat blast, caused by *Magnaporthe oryzae* pathotype *Triticum* (MoT), is a major constraint to global wheat production, with reported yield losses of up to 100% under highly conducive environmental conditions [1,2,3]. The effective management of this disease remains challenging mainly due to the lack of durable blast-resistant wheat varieties. Although BARI Gom 33, a local Bangladeshi variety, has exhibited moderate resistance to wheat blast, this resistance has not been consistently stable over time, possibly because of high temperature and humidity, and genetic and pathotypic variability within MoT populations [4,5].

Chemical fungicides are currently considered one of the most practical and effective approach for combating wheat blast [6,7]. However, the repeated and indiscriminate application of fungicides has contributed to accelerating the emergence of resistant pathogen populations, in addition to causing environmental contamination and disrupting beneficial microbial communities in the soil [8,9]. Resistance against major fungicide groups, including strobilurins, triazoles, and carboxamides, has been reported in Brazilian MoT populations, primarily due to their single-site modes of action [10,11,12]. Consequently, there is an urgent need for alternative, environmentally sustainable, and long-term disease management strategies to mitigate the impact of this devastating pathogen.

Plant-associated beneficial bacteria have gained considerable attention as eco-friendly biocontrol agents. These bacteria suppress pathogens through direct mechanisms, such as antimicrobial metabolite production, and competition for nutrients and ecological niches, as well as induction of plant defense responses through indirect mechanisms [13]. Compared with chemical fungicides, biocontrol agents exert lower environmental pressure and reduce the selection pressure for resistance development. Among them, *Bacillus* spp. have attracted significant interest due to their ability to produce a diverse range of antimicrobial compounds and promote plant growth [14,15,16,17]. In our previous studies, *Bacillus* strains, namely *B. subtilis* BTS-3, *B. velezensis* BTS-4, and *B. velezensis* BTLK6A, demonstrated strong antagonistic activity against MoT. Seed priming and foliar application of indigenous *Bacillus* strains significantly suppressed wheat blast in field conditions [18,19]. Given the demonstrated efficacy of these indigenous strains, the next critical step is the development of ideal and protective formulations for field application.

Maintaining high cell density and viability during both production and storage is vital for the efficacy of microbial biopesticides [20]. Profuse bacterial biomass depends on the selection of suitable fermentable growth media. In general, nutrient broth (NB), along with various salts are being used as growth media for bacterial isolates. Despite the fact that these commercial media are inextricably effective and widely used, they increase the capital cost required for production. Utilization of various agricultural-by-products offers an ecologically sustainable solution to natural waste management and production cost reduction. The most common and widely used by-products include molasses, rice bran, wheat bran, oat bran, peel of fruits and vegetables, corn liquor, etc., for bacterial fermentation [21,22,23]. Additionally, a suitable carrier material is another key factor for successful bacterial inoculants. The formulation systems help to maintain high viability and prolonged shelf life during storage and application. For dry formulation, talc and peat soils are commonly used as carrier materials whereas liquid bioformulations such as NB media, various plant-based oils, liquid molasses, sorbitol, etc., are used on a large scale for the management of plant diseases [24,25]. However, high-density viable cells of microbial inoculants in carrier often determines whether promising laboratory strains can be translated into economically viable products for crop production and protection. Therefore, formulation development has become a critical step in the advancement of microbial biocontrol technologies.

Despite their promise, developed bioformulations require extensive laboratory, greenhouse, and field evaluations before commercialization. Although many bioagents perform well under controlled pot experiments, their efficacy often declines or becomes inconsistent under field conditions [26,27,28]. The heterogeneous nature of soil ecosystems is one of the critical factors that limits the successful establishment of introduced microorganisms, as suitable ecological niches may already be occupied by indigenous microbial communities [28]. For successful field application, the inoculated strain must effectively colonize the rhizosphere and phyllosphere, compete with native microorganisms, and maintain a stable population over time. However, several studies have reported a rapid decline in bacterial inoculant within weeks after application, reducing their persistence and efficacy [29,30]. To compensate for these limitations, formulations often contain high initial cell densities to ensure that sufficient viable cells remain after storage and field application [31,32]. The endospore-forming ability of *Bacillus* spp. provides a major advantage by preserving viability during stabilization, storage and field application, thereby improving shelf life and environmental persistence [33]. Although bacterial populations gradually decline over time, well-formulated *Bacillus* inoculants retain sufficient viable bacterial cells that can still provide beneficial effects on plant growth and health under field conditions [31].

Based on our previous findings, the present study aimed to develop a *Bacillus*-based biopesticide formulation for wheat blast management. To address key challenges associated with large-scale production and commercialization, we first evaluated molasses as a low-cost and nutrient-rich agro-industrial by-product, as an alternative growth medium to optimize fermentation and maximize endospore production. Subsequently, we investigated and compared talc-based and liquid formulations for their ability to maintain bacterial viability and improve shelf-life stability during storage. Finally, we assessed the efficacy of the formulations against wheat blast under greenhouse and field conditions using different application methods. By replacing conventional nutrient broth with sugarcane molasses, this study bridges the gap between laboratory discovery and field application by developing an economically viable, shelf-stable *Bacillus*-based bioformulation with demonstrated efficiency against wheat blast.

## 2. Materials and Methods

### 2.1. Bacterial Strain and Culture Maintenance

*B. subtilis* BTS-3 (NCBI accession WOVJ00000000), *B. velezensis* BTS-4 (NCBI accession WOVK00000000), and *B. velezensis* BTLK6A (NCBI accession WOYD00000000) were obtained from the culture collection of the Institute of Biotechnology and Genetic Engineering (IBGE), Gazipur Agricultural University, Gazipur, Bangladesh [19]. All strains were routinely maintained in nutrient broth (NB; Liofilchem, Roseto degli Abruzzi, Italy) and preserved in 20% (*v*/*v*) glycerol stocks at −80 °C for long-term storage. Working cultures were revived on nutrient agar and incubated at 30 ± 2 °C under shaking conditions (150 rpm) for 24–48 h.

### 2.2. Effects of Growth Medium for Vegetative Growth and Spore Production

Commercial nutrient broth (NB; Liofilchem, Italy) and sugarcane molasses were evaluated as fermentation media for vegetative growth and endospore production of *Bacillus* spp. Food-grade sugarcane molasses was obtained from a sugar mill (Narsingdi, Dhaka, Bangladesh) and prepared at concentrations of 1%, 3%, 5% and 10% (*w*/*v*). The pH of each medium was adjusted to 7.0 prior to sterilization. Molasses solutions were filtered through sterile muslin cloth to remove insoluble particles and sterilized by autoclaving at 121 °C for 15 min. Commercial NB was prepared according to the manufacturer’s instructions and sterilized under identical conditions.

For vegetative growth, fresh 24 h bacterial cultures grown in NB were adjusted to approximately 1 × 10^8^ CFU (colony forming unit) mL^−1^, and 1% (*v*/*v*) inoculum was transferred into 50 mL of sterile medium contained in 100 mL Erlenmeyer flasks. Cultures were incubated at 30 ± 2 °C on a rotary shaker at 120 rpm for 24 h. Bacterial growth was determined by measuring the optical density at 600 nm (OD_600_), and the medium producing the highest biomass was selected for subsequent endospore production experiments [34].

Initially, endospore production was evaluated in the selected molasses-based medium and commercial NB without the addition of sporulation-promoting salts. Cultures were incubated under identical conditions, and preliminary observations at 24 and 48 h indicated limited endospore formation. The incubation period was therefore extended, and endospore production was subsequently assessed at 3, 6, and 9 days to characterize the relationship between fermentation duration and endospore yield and to determine a suitable working incubation period. Subsequently, the effects of NaCl, MgCl_2_·6H_2_O, ZnSO_4_·7H_2_O, or MnCl_2_·4H_2_O as sporulation-promoting salts on endospore production were evaluated individually in the selected molasses-based medium and commercial NB at a concentration of 500 ppm [35]. Based on preliminary concentration screening at 250, 500 and 750 ppm, 500 ppm was selected as the common concentration for further experiments, as the higher concentration resulted in reduced endospore production. Salt-supplemented cultures were incubated under the same conditions and sampled at the same time intervals (3, 6, and 9 days) for comparison.

Viable bacterial populations were enumerated by the spread plate method on nutrient agar (NA). Serial dilutions (1 × 10^5^–10^7^) were prepared, and 20 μL aliquots were spread onto six replicate NA plates per treatment, followed by incubation at 30 ± 2 °C for 24 h. Colony counts were used to calculate the viable bacterial population (CFU mL^−1^), which was log_10_-transformed and expressed as log_10_ CFU mL^−1^ for statistical analysis. For endospore enumeration, a 10 mL aliquot of each culture was heat-treated at 80 °C for 15 min to eliminate vegetative cells, followed by serial dilution (1 × 10^5^–10^7^) in sterile distilled water. Aliquots of 20 μL were plated on NA plates per treatment, followed by incubation at 30 ± 2 °C for 24 h. The sporulation rate was expressed as the percentage of heat-resistant spores relative to the viable cell count before heat treatment and calculated as follows:

$$\mathrm{Sporulation}\;\mathrm{rate}\;(\%)\;=\;\frac{\mathrm{CFU}{\mathrm{mL}}^{-1}\;\mathrm{after}\;\mathrm{heat}\;\mathrm{treatment}}{\mathrm{CFU}{\mathrm{mL}}^{-1}\;\mathrm{before}\;\mathrm{heat}\;\mathrm{treatment}}\times 100$$

The sporulation rate was used as an assessment criterion during the preliminary screening and the subsequent experiments focused on endospore production based on CFU mL^−1^.

### 2.3. Bioformulation Preparation

#### 2.3.1. Talc-Based Formulation

Talc-based formulations were prepared according to Sharifi-Tehrani et al. [36] with some modifications. Each endospore-forming bacterial strain was inoculated into 500 mL of sterile molasses broth (pH 7.0) in 1 L Erlenmeyer flasks (50% working volume to ensure aerobic conditions) and incubated at 30 ± 2 °C on a rotary shaker (150 rpm) for 72 h (until late stationary phase), yielding approximately 1 × 10^8^ CFU mL^−1^. Cells were harvested by centrifugation (8000× *g* for 10 min, 4 °C) and the pellet containing vegetative cells and endospores was resuspended in 30 mL sterile peptone water containing 5% (*v*/*v*) glycerol and incubated for 2 h at 150 rpm.

Sterile talc powder (100 g per strain), carboxymethyl cellulose (CMC; 1%, *w*/*w*), calcium carbonate (CaCO_3_; 1.5%, *w*/*w*) were sterilized twice by autoclaving at 121 °C for 15 min, cooled to room temperature (25 ± 2 °C), and then amended with the bacterial suspension and mixed thoroughly under aseptic conditions inside a laminar airflow cabinet [37].

The formulation was air-dried aseptically until a free-flowing powder was obtained, packaged in sterile polyethylene bags and stored at room temperature. Fresh formulations contained an initial viable cell count of ≥5 × 10^8^ CFU g^−1^. An overall procedure used for the preparation of the talc-based bacterial dry formulations, including the major processing steps, is schematically illustrated in Figure 1. For field experiments, bacterial biomass was produced in a 10 L pilot-scale bioreactor (with a 6–8 working volume) under identical culture conditions prior to formulation.

#### 2.3.2. Liquid Formulation

Liquid formulations were prepared using a protective base medium. Briefly, bacterial cultures were grown in 150 mL broth in a 250 mL bottle for 48 h at 30 ± 2 °C under shaking conditions (150 rpm) to reach approximately ≥5 × 10^8^ CFU mL^−1^. The formulation base (50 mL) consisted of glycerol (5%, *v*/*v*), trehalose (10 mM), sodium benzoate (0.1%, *w*/*v*), polyvinylpyrrolidone (PVP; 1%, *w*/*v*), PBS buffer and Tween 20 (0.1%, *v*/*v*) in distilled water; pH 7.00 was maintained using 1N NaOH before sterilization [38]. The base solution was prepared separately and sterilized by autoclaving at 121 °C for 15 min, followed by cooling to room temperature. After reaching the desired growth phase, the bacterial culture was aseptically mixed with the sterile formulation base to obtain a final volume of 200 mL inside a laminar airflow cabinet. The formulation was transferred to sterile airtight amber colored bottles, sealed and stored at room temperature (25 ± 2 °C) until further use. Two liquid formulations were prepared for each strain using (i) commercial nutrient broth and (ii) 1% molasses+ salt (MnCl_2_·4H_2_O) as the fermentation broth, while the composition of the protective formulation base remained identical in both liquid formulations. Based on shelf-life stability and viable cell retention, the molasses-based liquid formulation was selected for subsequent greenhouse and field validation experiments.

### 2.4. Shelf-Life Assessment of Formulated Bacterial Strains

To ensure standardized comparative analysis, all formulations were monitored for 180 days (6 months) under controlled storage conditions at room temperature (25 ± 2 °C). For solid formulations, 1 g samples were suspended in 10 mL sterile distilled water and serially diluted. For liquid formulations, 1 mL was added to 9 mL sterile distilled water prior to serial dilution. Aliquots (20 µL) were spread-plated on nutrient agar (NA) using six replicate plates per sample and incubated at 30 ± 2 °C for 24 h. Colony counts were used to calculate the viable bacterial population according to Section 2.2. Sampling was conducted at 0, 30, 60, 90, 120, 180 days after formulation.

### 2.5. Efficacy of Bioformulations Against Wheat Blast at Seedling Stage

The efficacy of bacterial bioformulations against wheat blast at seedling stage was evaluated under greenhouse conditions with three replications. Seeds of wheat cultivar BARI Gom 26 were treated with talc-based (Tlc) and molasses (Mls) liquid formulations of BTS-3, BTS-4, BTLK6A, and the consortium of these three. Prior to applying the combined bacterial formulation, the compatibility of strains BTS-3, BTS-4, and BTLK6A was evaluated using cross-streak and agar diffusion assays.

Selected bioformulations were applied via seed priming (P), foliar spray (F), alongside a chemical fungicide (Nativo) as positive control and only MoT treated as negative control. The experiment was arranged in a completely randomized design with three replications: 25 seeds were sown in each pot and 20 seedlings were kept by removing the other seedlings. Data from individual plants within each replicate were averaged, and the replicate mean was used as the experimental unit for statistical analysis.

Wheat seeds were bio-primed with the talc-based dry formulations at 2% (*w*/*w*) of the seed weight along with 2% glucose. The required amount of formulation and glucose were suspended in sterile distilled water and the seeds were kept with the suspension 12 h at room temperature, followed by 6 h of shade drying before sowing. The formulation was applied at a rate sufficient to achieve an approximate inoculum load of ≥1 × 10^7^ CFU seed^−1^.

For foliar treatments, a separate set of non-primed plants were used. The talc-based formulations were suspended in 2% glucose solution, incubated for 12 h (as previous), and sprayed onto the foliage 24 h before MoT inoculation. Untreated plants inoculated with MoT served as the negative control, while untreated uninoculated plants served as the healthy control. Nativo was included as the chemical standard control. Seedlings were artificially inoculated at 16 days after sowing by spraying a conidial suspension of MoT (1 × 10^5^ conidia mL^−1^) prepared from 7-day-old PDA cultures in sterile distilled water containing 0.01% Tween 20, onto the foliage. Following inoculation, plants were covered with transparent polyethylene sheets to maintain high humidity (>90% RH) for 48 h to facilitate infection according to the method described by [18] and treatment efficacy was calculated relative to the negative control.

Disease incidence and severity were recorded at 7 days post inoculation (DAI) using a 0–5 disease rating scale, where 0 = no visible symptoms and 5 = complete necrosis of the leaf blade [18], disease severity (DS %) was calculated as follows:

$$\mathrm{DS}=\frac{\sum (n\times v)}{N\times V}\times 100$$

where *n* is the number of leaves in each disease category, *v* is the numerical score of the corresponding disease category, *N* is the total number of leaves assessed, and *V* is the maximum disease rating. Disease severity reduction (DSR%) was calculated relative to MoT inoculated untreated control using the following equation:

$$\mathrm{DSR}(\%)\;=\;\frac{{\mathrm{DS}}_{\mathrm{control}}-{\mathrm{DS}}_{\mathrm{treatment}}}{{\mathrm{DS}}_{\mathrm{control}}}\times 100$$

### 2.6. Field Evaluation of Bioformulation Efficacy Against Wheat Blast

A field experiment was conducted at the research field of IBGE, Gazipur Agricultural University, Bangladesh, using a randomized complete block design (RCBD) with three replications. Each plot measured 1 m × 1 m and consisted of three rows spaced 0.20 apart, with 15 plants maintained per plot. Well-decomposed cow dung was applied at 5 tons ha^−1^ during land preparation. Recommended fertilizers (N-P-K-S at 70–28–50–11 kg ha^−1^) were applied according to the Fertilizer Recommendation Guide [39]. Two-thirds of urea and the full doses of all other fertilizers were applied as basal dose 3–4 days before sowing, while the remaining one-third of urea was top-dressed 20 days after sowing. The wheat cultivar BARI Gom 26, which is susceptible to wheat blast, was used throughout the study. Standard agronomic practices, including irrigation and intercultural operations, were performed as required. Environmental conditions during the experimental period were conducive to wheat blast development.

Bioformulations, application methods, and control treatments were identical to those described in Section 2.5. Seed-priming treatments were applied before sowing, whereas foliar treatments were applied 24 h before MoT inoculation. Artificial inoculation with MoT was performed at approximately 50% anthesis (flowering) following the standardized head inoculation protocol [18]. A conidial suspension (≥1 × 10^5^ conidia mL^−1^) prepared in sterile distilled water containing 0.01% Tween 20 was applied at approximately 0.75 mL spike^−1^, after which the inoculated plots were covered with moistened transparent polyethylene sheets for 48 h to maintain high humidity [40].

Disease incidence and severity were recorded at 14 days after inoculation (DAI). Disease severity (head blast index) was assessed using a 0–4 rating disease rating scale based on the percentage of infected spikelets, where 0 = no symptoms, 1 = 1–25.0%, 2 = 25.1–50.0%, 3 = 50.1–75.0%, and 4 = 75.1–100% infected spikelets [41]. Disease severity (%) was calculated as described in Section 2.5, and disease severity reduction (%) was determined relative to the MoT-only negative control. Agronomic parameters, including tiller number (recorded only for seed-priming treatments), thousand-grain weight, and grain yield per plot, were measured following standard procedures. The relative increase in effective tiller number, total grain yield (g plot^−1^), grain yield (%), and thousand-grain weight (%), calculated with respect to the negative control.

### 2.7. Statistical Analysis

Bacterial viability and sporulation experiments were conducted using six plate replicates per treatment. Colony-forming unit (CFU) data were log_10_-transformed before analysis to satisfy the assumptions of normality and homogeneity of variance. Depending on the experimental design, one-, two-, or three-way analysis of variance (ANOVA) was performed to evaluate the effects of formulation, bacterial strain, storage period, culture medium, and their interactions.

Greenhouse and field experiments were arranged in a randomized complete block design (RCBD) with three biological replicates per treatment. Disease severity reduction (DSR), growth parameters, and yield-related traits were analyzed using a three-way factorial ANOVA to determine the effects of bacterial strain (B), formulation type (F), application method (M), and their interactions (B × F, B × M, F × M, and B × F × M). When significant treatment effects were detected, means were compared using Tukey’s HSD test at *p* ≤ 0.05. Data are presented as mean ± standard error (SE) unless otherwise stated. Graphs were prepared using the ggplot2 package in R (version 4.5.1; R Core Team, Vienna, Austria).

## 3. Results

### 3.1. Effect of Molasses Concentration on Vegetative Growth and Endospore Production of Bacillus *spp.*

To reduce the fermentation costs, sugarcane molasses was tested as a cheap fermentative source of *Bacillus* spp. The effect of molasses concentration on the growth of three *Bacillus* strains was evaluated using two-way ANOVA based on log_10_ CFU. Sugarcane molasses supported significant growth (*p* < 0.001) of all three *Bacillus* strains; however, growth performance differed significantly among the strains (*p* < 0.001) and the strain–molasses interaction (*p* < 0.001) indicated strain-specific response (Table 1).

Among the tested concentrations, 1% molasses supported bacterial growth comparable to or slightly higher than that obtained in commercial NB (Figure 2) across all *Bacillus* strains. Low molasses concentrations (1–3%) generally supported higher colony counts than 5–10% molasses. In 1% molasses, BTS-3, BTS-4 and BTLK6A showed high cell densities, reaching 9.90 ± 0.03, 9.71 ± 0.02, 10.08 ± 0.01 log_10_mL^−1^, respectively, which were equivalent to or marginally higher than their growth in NB (9.63 ± 0.01 log_10_mL^−1^). Although, BTS-4 exhibited its highest growth at 3% molasses, yielding 9.85 ± 0.01 log_10_mL^−1^, its performance in 1% molasses still remained comparable to NB. Increasing molasses concentration beyond 3% progressively reduced bacterial growth in all isolates, with the lowest colony count recorded at 10% molasses (Figure 2).

### 3.2. Optimization of Culture Medium for Enhanced Endospore Production

#### 3.2.1. Effect of Molasses-Based Medium on Endospore Production Efficiency

Sporulation efficiency differed significantly among the culture media (*p* < 0.001) (Table S1). For BTS-3 and BTS-4, both molasses-based media produced significantly higher spore densities than NB throughout the incubation period, with 1% molasses consistently higher. At day 3, endospore counts in 1% molasses ranged from 6.87 to 8.61 log_10_ CFU mL^−1^ among all the three strains, compared with 6.65–8.48 log_10_ CFU mL^−1^ in 3% molasses and 5.27–8.36 log_10_ CFU mL^−1^ in NB. The same trend was maintained at later incubation periods, indicating that 1% molasses was more suitable for inducing sporulation (Figure 3A).

#### 3.2.2. Effect of Mineral Salts on Endospore Production Efficiency in 1% Molasses Medium

Supplementation of different mineral salts to 1% molasses medium influenced endospore production significantly across all strains. All the individual factors and their interactions contributed to the observed variation (*p* < 0.001) (Table S2).

Among the four tested salts, MnCl_2_·4H_2_O showed the highest effect on endospore production followed by MgCl_2_·6H_2_O and ZnSO_4_·7H_2_O, while NaCl showed lowest endospore production compared to other treatments. At 3 days of incubation, MnCl_2_·4H_2_O supplementation resulted in endospore production ranging from 6.94 to 8.95 log_10_ CFU mL^−1^, compared with 6.87–8.61 log_10_ CFU mL^−1^ in the 1%-only molasses medium. The positive effect of MnCl_2_.4H_2_O was maintained throughout incubation, with spore densities ranging from 6.98 to 8.99 and 6.96 to 8.91 log_10_ CFU mL^−1^ at day 6 and day 9, respectively (Figure 3B). Although sporulation increased up to day 6, further incubation did not substantially improve spore production.

Although sporulation increased slightly up to day 6, the additional increase after day 3 was limited; therefore, 1% molasses supplemented with 500 ppm MnCl_2_·4H_2_O and a 3-day incubation period were selected as the optimized fermentation conditions for subsequent bioformulation development, balancing high endospore production with reduced fermentation time, energy consumption, and operational costs.

#### 3.2.3. Cost Assessment of 1% Molasses-Based Fermentation Media

From an economic perspective, replacing NB with a molasses-based medium substantially reduced production costs relative to commercial NB. The estimated preparation cost of NB was approximately BDT321 per liter, whereas the molasses-based medium cost only BDT 102–103 per liter (Table S3). Thus, molasses supplementation reduced medium preparation costs by approximately BDT 218–219 per liter, corresponding to nearly 68% cost savings. The reduction was achieved by replacing expensive commercial nutrients with an inexpensive agro-industrial by-product while maintaining comparable bacterial growth and sporulation efficiency.

### 3.3. Effect of Formulation and Storage Duration on Bacillus Shelf-Life

The shelf-life of *Bacillus* strains during storage was strongly influenced by formulation type, storage duration, bacterial strain and their interactions (*p* < 0.001) (Table S4). All formulations exhibited comparable initial populations (8.22–8.94 log_10_ CFU) at day 0.

The talc-based dry and liquid formulations exhibited distinct survival patterns during the 180-day storage period (Figure 4). The talc-based formulation showed the highest stability, with viable count remaining 6.91–7.12 log_10_ CFU g^−1^ after six months for all strains (Figure 4A). In comparison, liquid formulation showed a rapid decline, with molasses-based formulations retaining 3.85–4.49 log_10_ CFU mL^−1^ and NB-based formulations retaining 2.72–3.65 log_10_ CFU mL^−1^ at day 180 (Figure 4B,C).

Although significant strain-dependent effects were observed, all *Bacillus* strains exhibited a comparable decline pattern throughout the storage period. BTLK6A generally retained higher viable populations across formulations, whereas BTS-3 and BTS-4 also maintained substantial survival, particularly in the talc-based formulation (Figure 4). The significant formulation × strain × storage interaction indicates that bacterial persistence was influenced by the combined effects of strain characteristics and the protective properties of formulation (Table S4).

### 3.4. Effects of Dry and Liquid Bioformulation on Seedling Stage

The compatibility assessment showed no obvious antagonistic interactions between BTS-3, BTS-4 and BTLK6A, supporting their combined application (Scheme S1).

Analysis of variance showed that application method significantly influenced disease severity reduction (DSR) (*p* = 0.005), but formulation type, bacterial strain and their interactions were non-significant (*p* > 0.05) (Table S5). At the seedling stage, disease severity reduction over negative control ranged from 69.40 ± 3.22% to 79.06 ± 3.22% among the treatments (Figure 5). Seed-priming treatments showed numerically higher disease suppression, with BTS-3 talc (79.06 ± 4.17%), BTS-4 talc (75.85 ± 4.85%); however, these numerical differences among bacterial strains and formulations were not statistically significant, as all treatment means shared the same Tukey’s HSD grouping.

### 3.5. Field Efficacy of Bioformulations Against Wheat Blast

#### 3.5.1. Field Efficacy of Bacillus Bioformulations Against Wheat Blast

Field evaluation showed noticeable differentiation among the treatments compared to the seedling assay. Disease reduction was significantly influenced by bacterial strains (*p* = 0.005) and application methods (*p* < 0.001) in the field but not the formulation types. Factorial ANOVA identified application method as the principal factor affecting field disease severity (F = 80.50, *p* < 0.001), whereas bacterial strains had a smaller but significant effect (F = 5.32, *p* < 0.01). The main effect of formulation and the three-way interaction were not significant (*p* > 0.05), indicating that the superiority of seed priming depended primarily on bacterial strains rather than formulation alone (Table S5).

At 14 DAI, disease severity across all the treatments ranged from 9.62 to 20.39%, compared with the negative control (39.90 ± 1.34%) and the positive control (Nativo) (4.27 ± 0.65%) (Table S6). Disease severity reduction ranged from 48.78% to 75.98% among the bacterial treatments (Figure 6). The highest disease suppression was achieved by BTS-4 talc-based seed priming (75.98 ± 1.68%), followed by BTS-3 talc-based priming (75.49 ± 1.80%), BTS-4 molasses-based priming (73.42 ± 2.91%), BTLK6A talc-based priming (68.16 ± 1.79%). Foliar applications generally produced lower disease suppression, ranging from 48.78 to 60.38% (Figure 6). Overall, seed priming consistently outperformed foliar application, with talc-based formulations of BTS-3 and BTS-4 providing higher disease suppression.

Representative field plots showing the disease response of wheat plants under different treatments are presented in Figure 7.

#### 3.5.2. *Bacillus*-Based Bioformulations Enhance Plant Tiller Number and Increase Yield

To test the effects of seed priming in plant growth promotion, all the agronomic data were compared among the treatments. Seed priming with *Bacillus*-based bioformulation, either talc- or liquid- based, significantly influenced effective tiller number increase under field conditions (Figure 8A,E). Two-way ANOVA revealed significant effects among the bacterial strains (*p* < 0.001), and formulation types (*p* = 0.007) but their interaction was not statistically significant (*p* = 0.781) (Table S7). The increase in effective tiller number of seed-primed plants over the non-primed control plants ranged from 9.40 ± 2.16% to 16.62 ± 0.76% and the highest effective tiller increase was recoded in BTLK6A talc-based seed priming. The molasses-based seed priming of consortium showed lowest effective tiller increase over non-primed control (Figure 8A).

For yield-related traits, formulation was the dominant factor, showing the strongest effects on thousand-grain weight (F = 178.31, *p* < 0.001) and relative yield increase (F = 104.59, *p* < 0.001). Bacterial strain had the strongest influence on total grain yield (F = 137.37, *p* < 0.001), while application method also significantly affected all yield traits. Nevertheless, the generally non-significant three-way interactions suggest that the main effects of formulation and bacterial strain remained robust across different treatment combinations (Table S5). The highest thousand-grain weight was recoded for BTS-4 seed priming with talc (32.41 ± 2.05%), followed by BTS-4 talc-based foliar application (29.71 ± 2.30%), while foliar spray of consortium in molasses formulation recorded the lowest increase (13.47 ± 0.61%) (Figure 8B). The highest total grain yield plot^−1^ was obtained by BTS-4 talc-based seed priming (321.00 ± 4.16 g), followed by BTLK6A talc-based seed priming (313.00 ± 3.06 g) and BTS-3 talc-based seed priming (303.33 ± 3.18 g). The lowest yield was recorded for consortium molasses-based seed priming (260.00 ± 3.61 g). The healthy control (352.33 ± 4.91 g) and the fungicide treatment (337.67 ± 2.40 g) remained superior to all bacterial treatments, whereas the inoculated untreated control produced the lowest yield plot^−1^ (231.67 ± 2.73 g) (Figure 8C). Therefore, relative yield grain increased by 12.23–38.58%, with the highest value recorded for BTS-4 seed priming with talc formulation (38.58 ± 1.64%), followed by BTLK6A talc-based seed priming (35.16 ± 2.48%) (Figure 8D).

## 4. Discussion

The rapidly increasing threat of wheat blast and its implications for global food and nutritional security have intensified the demand for sustainable disease-management strategies. Within integrated pest management (IPM), biological control using endophytic bacteria offers promising, environmentally safer biotechnology alternatives to synthetic fungicides [42]. Although BTS-3, BTS-4 and BTLK6A previously showed strong antagonism against MoT [15,18,19], their practical application depends on cost-effective production systems and formulations capable of maintaining microbial viability, stability and field efficacy during storage [43]. The present study demonstrated that integrating low-cost fermentation media, optimized endospore production, stable carrier formulations, and appropriate application methods enable endophytic *Bacillus* strains (BTS-3, BTS-4, and BTLK6A) to be developed into practical biocontrol agents for wheat blast management, with BTS-4 consistently exhibiting the highest field efficacy.

A key finding of this study is that sugarcane molasses can effectively replace commercial nutrient broth as an economical fermentation substrate for *Bacillus* biomass production. Keeping production cost low is a key factor to making microbial inputs commercially viable and agriculturally profitable. Sugarcane molasses, a readily available by-product of the sugar industry in Bangladesh, offers an effective alternative as carbon source for bacterial cultivation [44]. The present study demonstrated that 1–3% molasses supported bacterial vegetative growth and endospore production comparable to commercial nutrient broth. The performance of low-concentration molasses is likely attributable to its balanced supply of substantial amounts of fermentable sugars (46–52%) [45], amino acids, essential trace minerals (SO_4_^2−^, Ca^2+^, Na^+^, etc.,) and vitamins that provide a nutrient-rich environment to promote robust bacterial growth due to metabolic efficiency which supports not only vegetative growth but also induce sporulation [24,34,46,47]. Therefore, utilization of sugarcane molasses as a fermentation substrate contributes to the development of more affordable microbial inputs beyond laboratory use while supporting sustainable agricultural practice.

Endospore formation is critical for *Bacillus*-based products as spores exhibit higher resistance to desiccation, temperature fluctuation and storage-associated stress far better than vegetative cells. Although *Bacillus* spp. are naturally endospore-forming, sporulation efficiency varies considerably among strains [48,49,50,51]. Among the tested isolates, all isolates responded strongly in molasses-based medium, particularly in the presence of MnCl_2_·4H_2_O. This observation is consistent with the well-established role of Mn^2+^ in *Bacillus* sporulation, where manganese serves as an essential cofactor regulating the transition from vegetative growth to endospore formation [52,53]. Enhanced sporulation is particularly advantageous for commercial formulations while ensuring longer shelf life and field persistence.

The choice of carrier material strongly influenced long-term bacterial survival. Among the evaluated carriers, talc-based formulation consistently maintained the highest viable cell count and demonstrated a standard cell count (approx. 1 × 10^6^–10^7^ CFU g^−1^) up to 180 days. A similar finding was reported by Novinscak and Filion [30]. Talc likely improves shelf life by reducing water activity, limiting metabolic activity, and protecting cells from stress [37,54]. The survival and shelf life of *Bacillus* declines rapidly in molasses and NB-based liquid formulations because metabolically active cells gradually exhaust available nutrients and energy reserves, undergo starvation, and die [25]. The prolonged viability in talc-based formulations observed in this study may also be attributed to the protective effects of CMC, glycerol, and peptone, which function as osmo-protectants and reduce stress during storage.

Seed biopriming proved to be the most effective and sustainable technique for the efficient use of antagonistic microorganisms to enhance crop productivity under stressful environment [55,56]. Previous studies on BTS-3, BTS-4 and BTLK6A have demonstrated strong antagonistic activity against the wheat blast pathogen (MoT) and also identified genomic features of these bacteria that are potentially associated with the biosynthesis of antimicrobial metabolites, such as fengycin, iturin, surfactin, etc., [19]. Subsequent GC-MS analysis further detected volatile organic compounds (VOCs), including phenylethyl alcohol, hexanoic acid, 2-methylbutanoic acid, acetoin, hexanol, and 2,3-butanediol, while in vitro assays with selected pure compounds confirmed their direct role in pathogen suppression [14], consistent with a previous field study in which BTS-4 showed particularly strong field performance [18]. The comparatively strong field performance of BTS-4 may reflect the combined effects of multiple strain-specific biocontrol characteristics rather than endospore production capacity alone. Since BTLK6A exhibited higher endospore production than BTS-4 in the present study, strain-specific factors, including antimicrobial activity, VOC-mediated pathogen suppression, plant-associated persistence, colonization, or adaptation from laboratory conditions to field conditions, may contribute to differential field efficiency. Volatile profiling of these three isolates revealed that BTS-4 produced 12 unique VOCs compared to other strains [14], among which acetoin and 3-pentanol are well recognized for their antifungal and induced systemic resistance properties [57,58]. It has been reported that increased acetoin production by the engineered *B. velezensis* strain GJ11Δ*bdh* could trigger much stronger induced systemic resistance (ISR) than the wild-type strain GJ11. However, other physiological parameters, such as pH, temperature, and colonization ability need to be tested to better understand the underlying mechanism of the superior performance of BTS-4.

In the current study, the consistently superior performance of seed priming with the bacterial isolates is most plausibly driven by early tissue colonization that establishes a durable, immune-primed state. This priming is mediated by microbial elicitor perception, ISR activation, JA/ET-dependent signaling, MAPK cascades, and the transcriptional reprogramming of defense-related genes. Upon pathogen challenge, bio-primed plants exhibit rapid ROS accumulation, PR gene induction, elevated defense enzyme activities, and metabolomic shifts that effectively restrict fungal colonization [59,60,61]. Emerging evidence indicates that such long-term protection also involves epigenetic regulation, including altered DNA methylation and enhanced chromatin accessibility at defense loci, as previously demonstrated for *Bacillus*-mediated resistance against *Bipolaris sorokiniana* [62]. Conversely, disease suppression via foliar sprays relies primarily on localized mechanisms. These include direct antagonism through the secretion of lipopeptides and extracellular lytic enzymes, as well as localized pattern-recognition receptor (PRR) activation. While foliar applications can trigger JA/ET- or SA-mediated signaling, PR protein expression, and cell wall reinforcement upon pathogen contact, this localized response provides more transient protection compared to the systemic, persistent immunity conferred by seed priming [26,63]. Similar long-term stability of talc-based *Bacillus* formulations has been reported against several plant diseases (41.3–73.3% suppression of rapeseed damping-off, up to 97.15% control of bacterial leaf blight and around 53% control of *Fusarium verticillioides*) with viability after 360 days [36,64,65].

Another key finding of this study is that, in addition to suppressing wheat blast, the formulated *Bacillus* strains significantly improved wheat growth and yield. Seed priming increased effective tiller number, thousand-grain weight, and grain yield compared with the pathogen-inoculated control. Reduced disease severity likely contributed directly to improved grain filling and yield by protecting spikes from MoT infection during grain development [3]. Additional yield benefits may result from improved nutrient acquisition and production of phytohormones [44,66,67,68]. Recent studies also suggest that *Bacillus* spp. produce a diverse range of cyclic dipeptides such as cyclo (L-Ala-L-Ile), cyclo (L-Leu-L-Pro) which act as biological elicitors to trigger plant growth by inducing and mimicking hormonal signaling [69,70]. Whether our *Bacillus* strains produce similar cyclic dipeptides needs to be investigated to elucidate the precise mechanisms underlying growth promotion.

In conclusion, this study highlights the dual functionality of optimized *Bacillus* bioformulations as both potent bio-pesticides and effective plant growth promoters. Talc-based seed priming with strains BTS-3 and BTS-4 provides a cost-effective, scalable, and environmentally sustainable alternative to synthetic chemicals. While chemical fungicides like Nativo^®^ offer rapid knock-down efficacy, biopriming provides durable, multi-layered immunity with long-term ecological benefits. To transition these findings from experimental trials to commercial agricultural deployment, multi-location field testing across diverse agro-climatic zones is now warranted to confirm the stability and reliability of these formulations under variable environmental conditions.

## Figures and Tables

**Figure 1:**
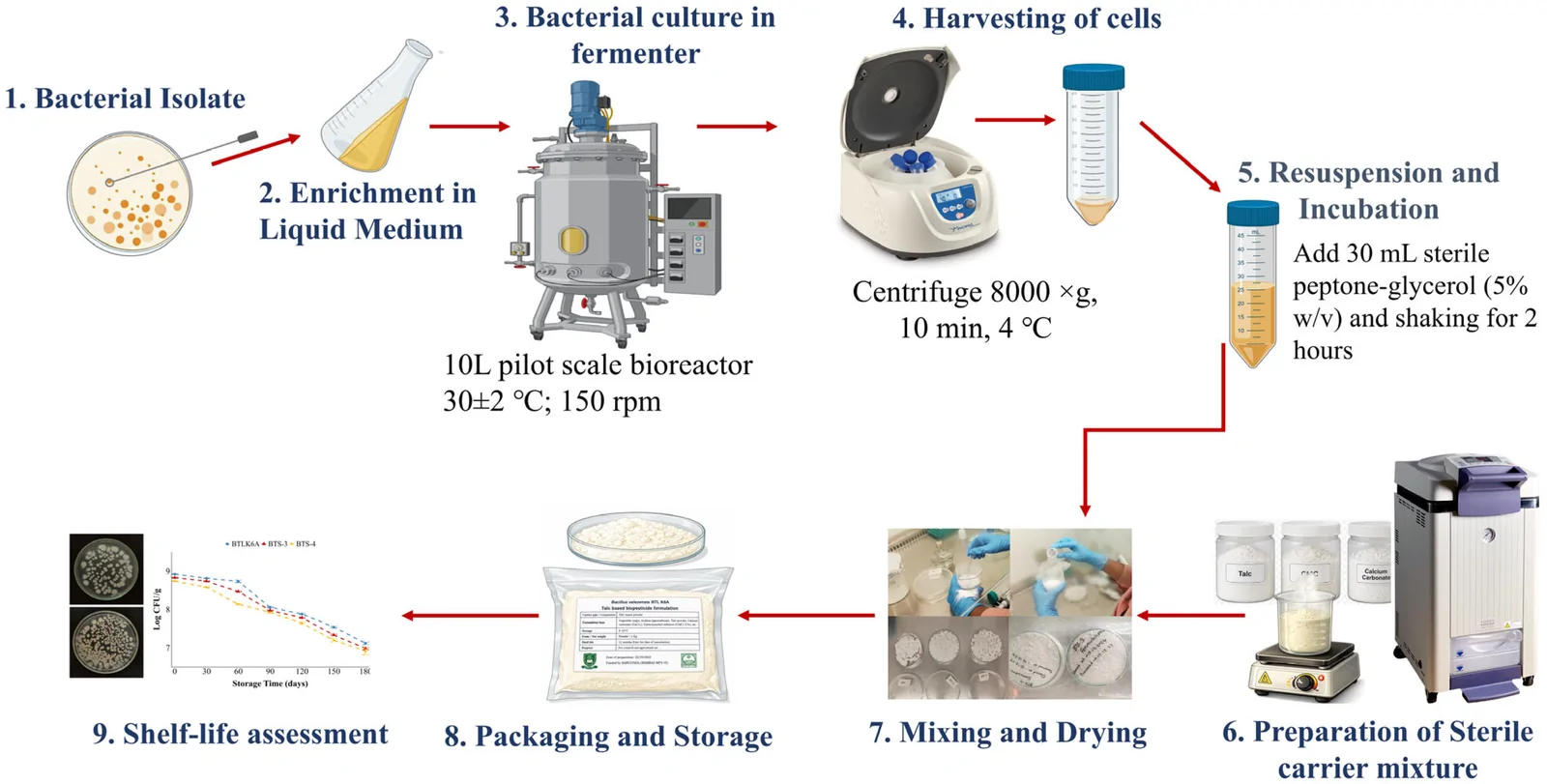
Schematic illustration for preparing talc-based bacterial dry formulations.

**Figure 2:**
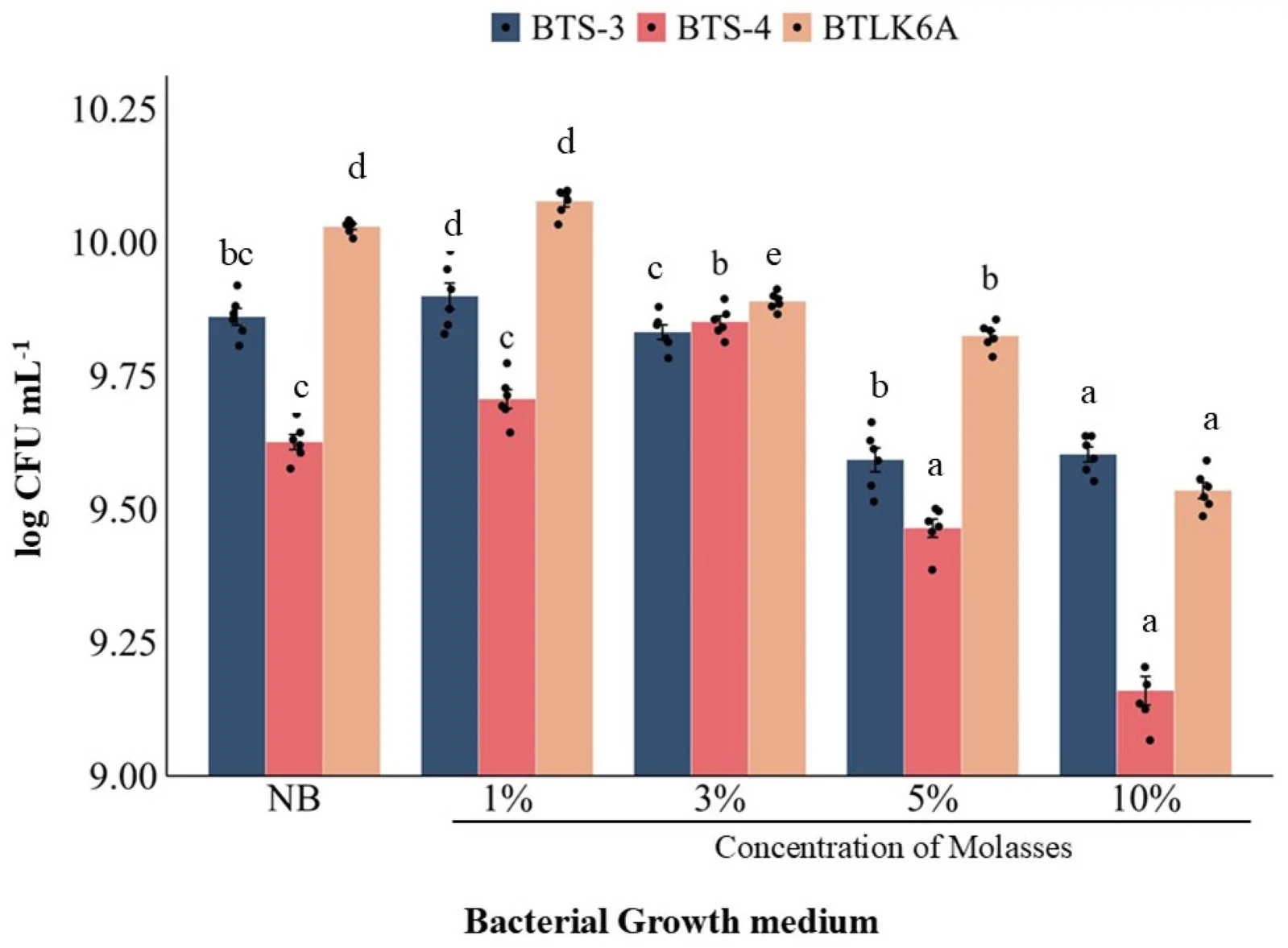
Growth performance of *Bacillus* strains in molasses-based liquid media. Vegetative cell counts (log_10_ CFU mL^−1^) of BTS-3, BTS-4 and BTLK6A cultured in NB and different concentrations of molasses after 24 h incubation. Low molasses concentrations (1–3%) supported comparable or improved growth relative to NB. Values represent mean ± SE of six biological replicates. Different letters indicate significant differences among molasses concentration within each bacterial strain according to Tukey’s HSD test (*p* ≤ 0.05), following two-way ANOVA.

**Figure 3:**
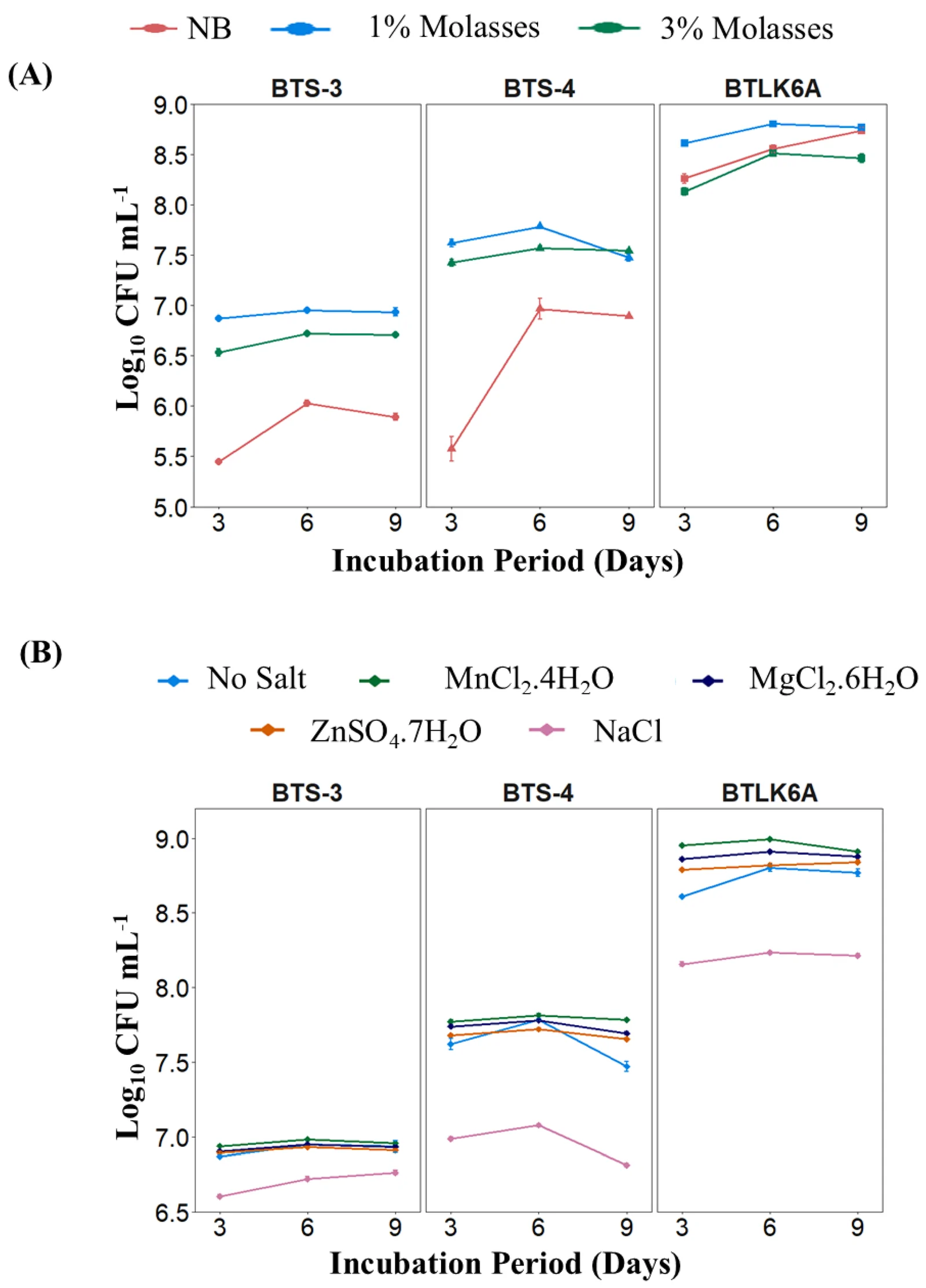
Effect of molasses-based growth medium for enhanced endospore production: (**A**) endospore dynamics in nutrient broth (NB), 1% molasses, and 3% molasses-based media, (**B**) effect of different salt amendments supplemented with 1% molasses medium on *Bacillus* sporulation efficiency. Data are presented as mean ± standard error (SE) of six plate replicates.

**Figure 4:**
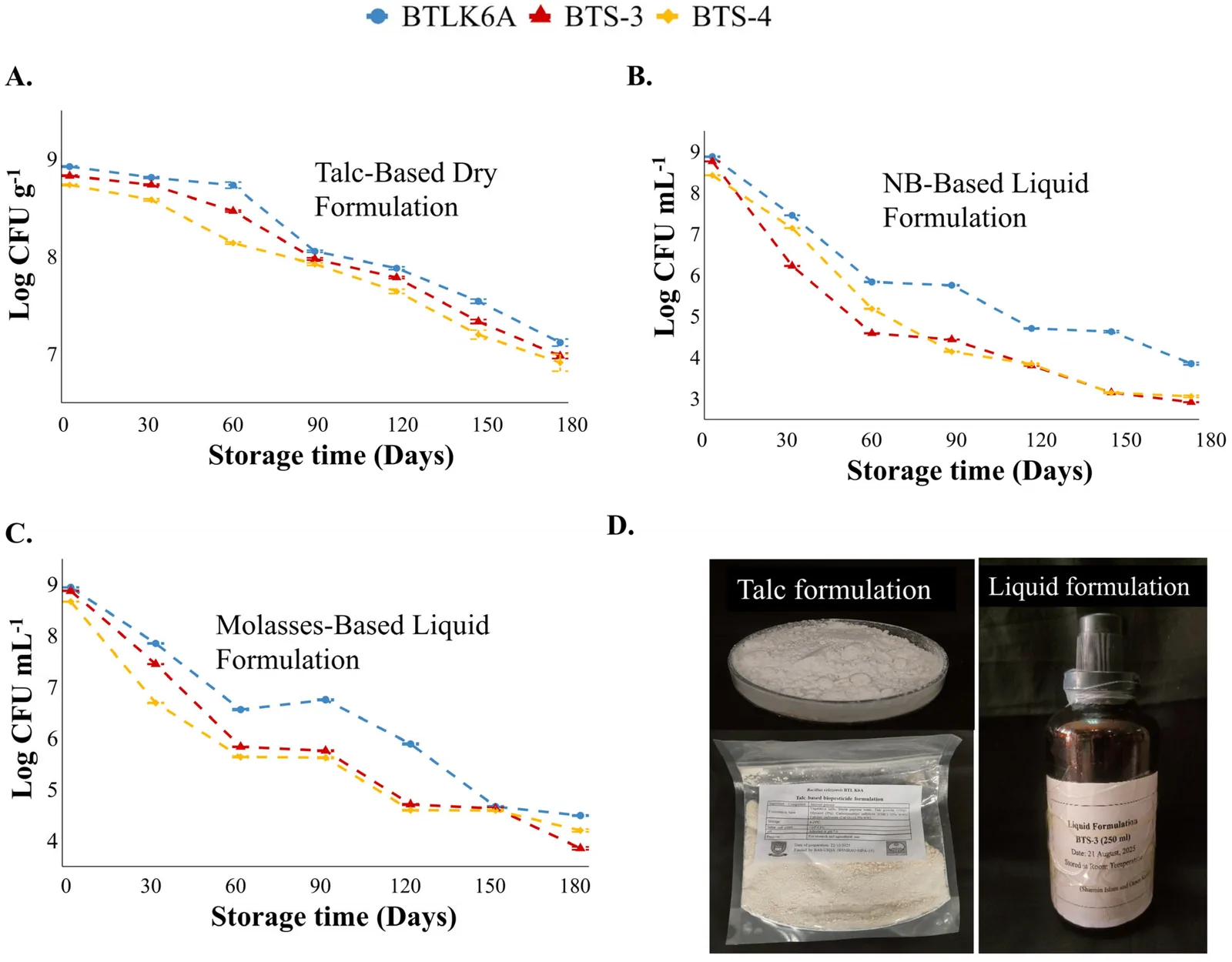
Shelf-life evaluation of BTS-3, BTS-4 and BTLK6A in different formulation types during storage. (**A**) Talc-based dry formulation, (**B**) NB-based liquid formulation, and (**C**) molasses-based liquid formulation, (**D**) packaging of the formulations. Data represent mean ± SE of six replicates (n = 6).

**Figure 5:**
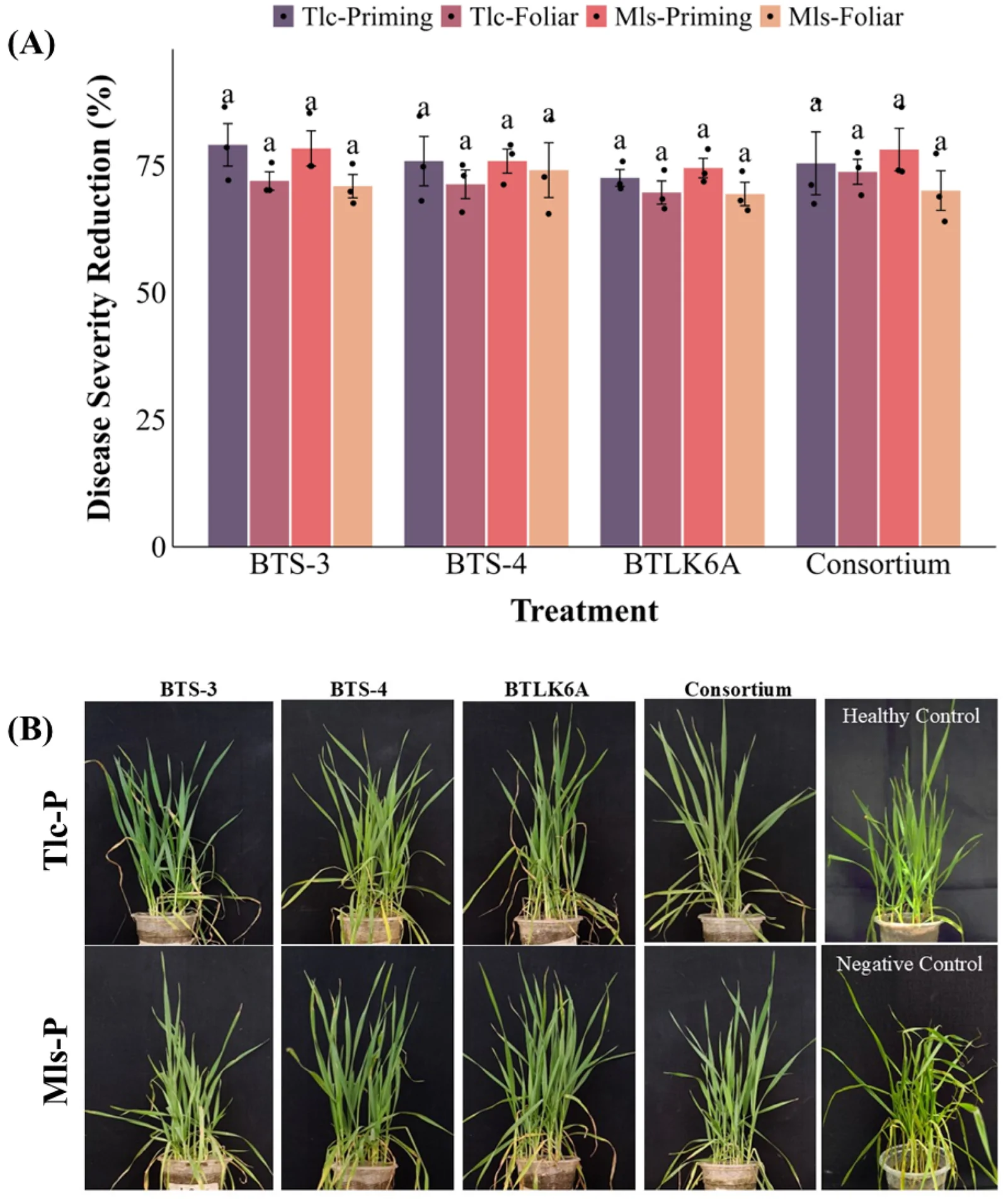
(**A**) Disease severity reduction (%) of wheat seedlings treated with *Bacillus* spp. BTS-3, BTS-4, BTLK6A, and their consortium applied through talc-based priming (Tlc-P), talc-based foliar spray (Tlc-F), molasses-based priming (Mls-P), and molasses-based foliar spray (Mls-F). (**B**) Representative images show bacterial seed priming with Tlc-P and Mls-P compared with healthy and negative controls. Bars represent mean ± SE (n = 3). The same letter indicates no significant differences among treatment means according to Tukey’s HSD test (*p* ≤ 0.05).

**Figure 6:**
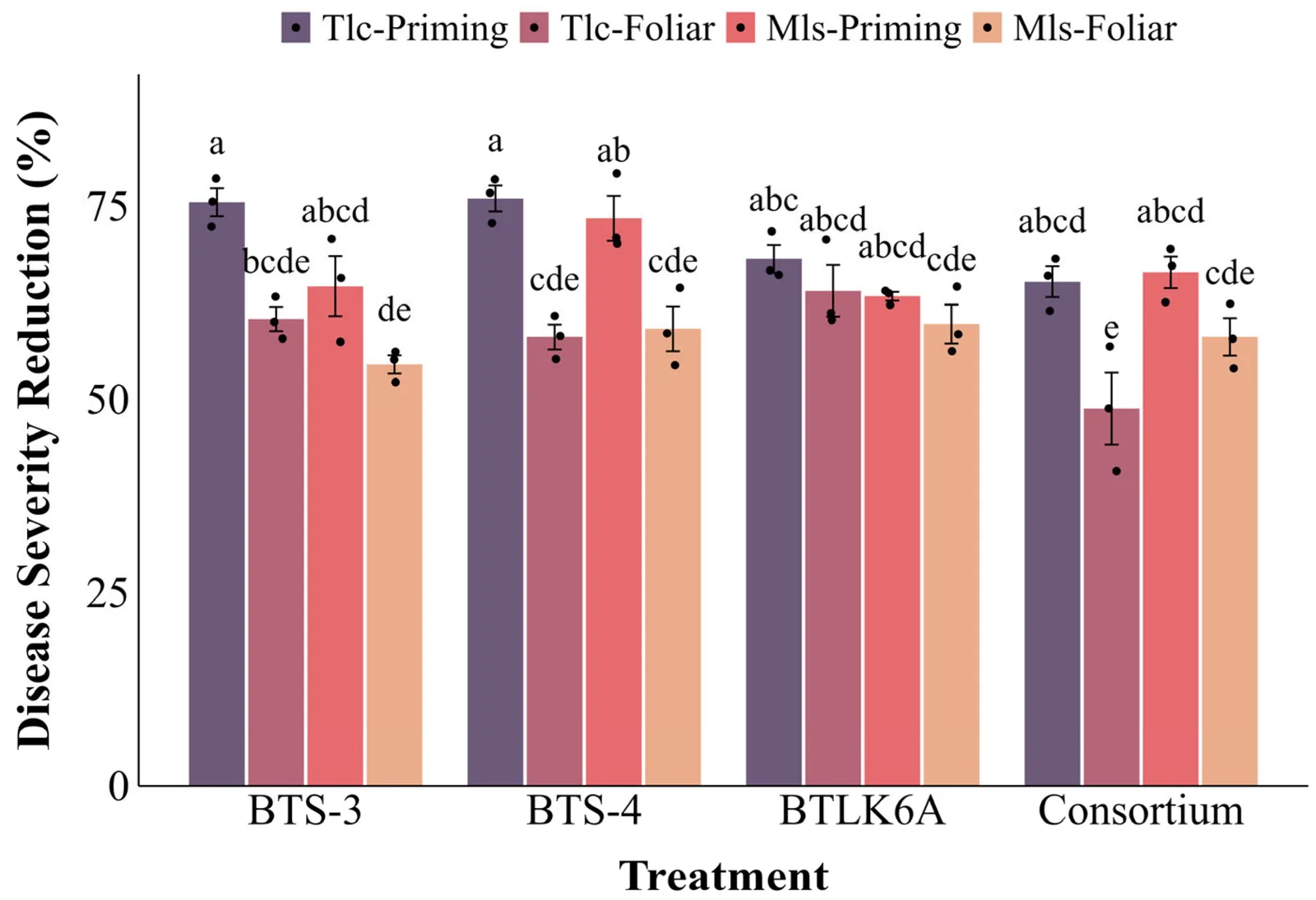
Disease severity reduction (%) in BARI Gom 26 under field condition treated with *Bacillus* spp. BTS-3, BTS-4, BTLK6A and consortium applied through talc-based priming (Tlc-P), talc-based foliar spray (Tlc-F), molasses-based priming (Mls-P), and molasses-based foliar spray (Mls-F). Bars represent mean ± SE (n = 3). Means sharing at least one common letter are not significantly different according to Tukey’s HSD test at *p* ≤ 0.05.

**Figure 7:**
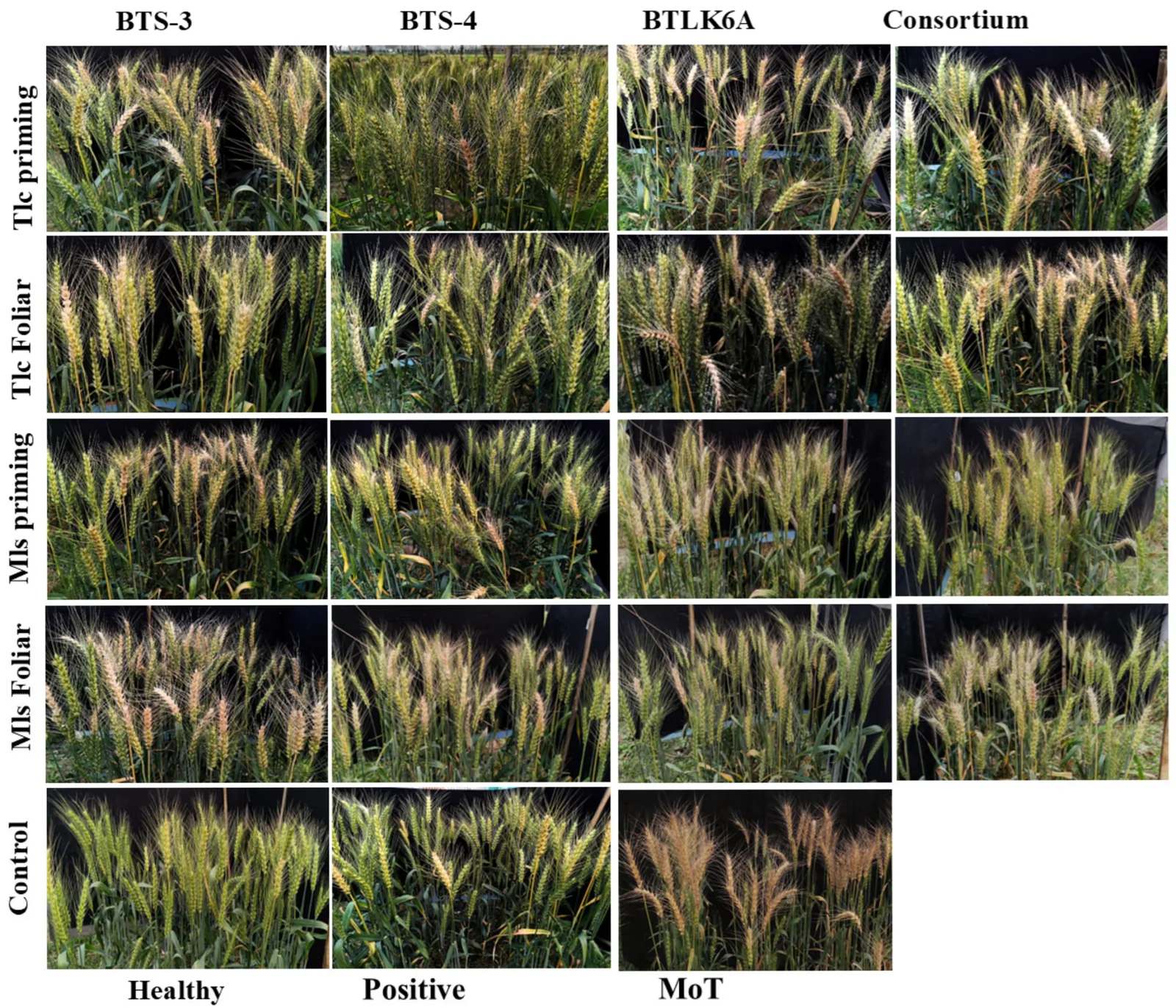
Representative photographs of wheat plants demonstrate phenotypic response (after 14 days of MoT inoculation) treated with three bacterial strains (BTS-3, BTS-4, BTLK6A, and their consortium) formulations.

**Figure 8:**
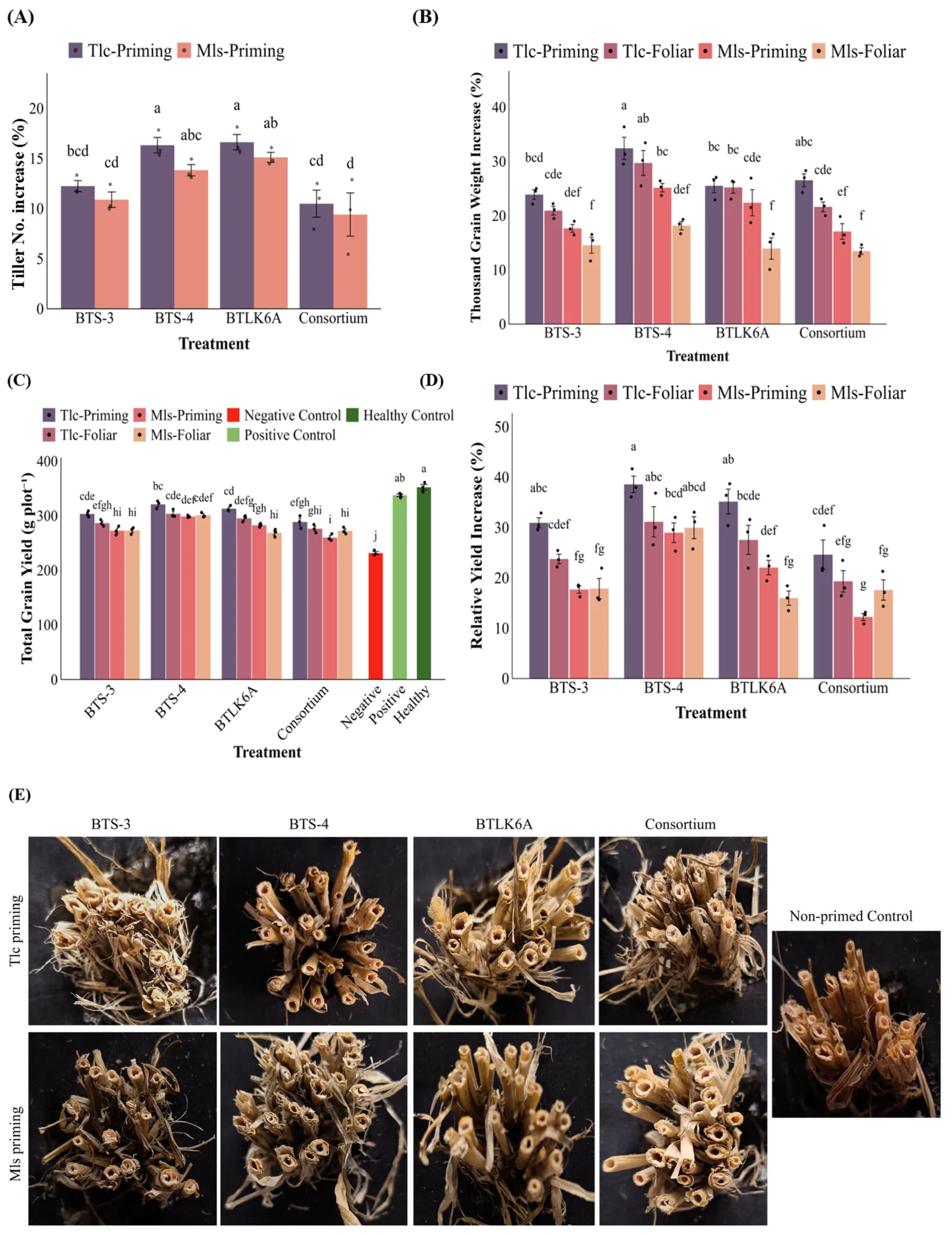
Effects of talc- and molasses-based *Bacillus* formulations applied through seed priming (P) and foliar application (F) on wheat growth and yield parameters under wheat blast infection. (**A**) Percent increase in tiller number over the control. (**B**) Percent increase in thousand-grain weight over the negative control. (**C**) Total grain yield per plot (g plot^-1^). (**D**) Grain yield increase (%) over the negative control. (**E**) Representative image of harvested wheat crowns corresponding to tiller number in the seed priming treatments. Bars represent mean ± SE (n = 3). Black dots indicate individual biological replicates. Different letters above the bars indicate significant differences among treatments according to Tukey’s HSD test (*p* ≤ 0.05).

**Table 1:** Two-way ANOVA for the effects of medium formulation, bacterial isolate, and their interaction on *Bacillus* population density.

Source of Variation	Df	Sum Sq	Mean Sq	F Value	Pr (>F)
Growth medium	4	2.772268	0.693067	442.9216	<0.001
Bacteria	2	1.471594	0.735797	470.2293	<0.001
Medium: Bacteria	8	0.526341	0.065793	42.04639	<0.001
Residuals	75	0.117357	0.001565	-	-

Note: Statistical significance was considered at *p* < 0.05. - indicates not applicable.

## Data Availability

Data will be made available upon valid request to the corresponding author.

## Supplementary Materials

The following supporting information can be downloaded at https://www.mdpi.com/article/10.3390/jof12100766/s1, Table S1. Three-way ANOVA for culture medium optimization medium on sporulation. Table S2. Three-way analysis of variance (ANOVA) for the effect of salt supplementation on bacterial sporulation. Table S3. Comparative cost for the preparation of low-cost culture media. Table S4. Effect of formulation, bacterial strain, and storage duration on *Bacillus* viability (three-way ANOVA). Analysis was performed on log-transformed viable bacterial population (log_10_ CFU). Table S5. Three-way ANOVA for disease suppression and yield-related parameters of wheat under field conditions. Table S6. Field disease severity of wheat blast under different bacterial treatments. Table S7. Two-way Analysis of variance for tiller number increase (%) by seed priming with *Bacillus*-formulation. Scheme S1. Compatibility of *Bacillus* strains BTS-3, BTS-4, and BTLK6A assessed by cross-streak plate and agar-based diffusion assays.

## Abbreviations

The following abbreviations are used in this manuscript:

IMP	Integrated pest management
ISR	Induced systemic resistance
VOC	Volatile organic compounds
GC-MS	Gas chromatography–mass spectrometry
MoT	*Magnaporthe oryzae Triticum*
CFU	Colony-forming unit
JA	Jasmonic acid
SA	Salicylic acid
PRR	Pattern recognition receptor
ET	Ethylene
