# Supplementary material for: Optimized Endospore Production and Carrier Formulations of Endophytic Bacillus Species to Enhance Field Control of Wheat Blast
Source: J Fungi (Basel). 2026 Oct 12;12(10):766. doi: 10.3390/jof12100766 (PMC13706097; doi:10.3390/jof12100766)
Supplement: Supplementary file 1 [file jof-12-00766-s001.zip › jof-4512227-supplementary.pdf]

Journal of *Fungi*

MDPI

Optimized Endospore Production and Carrier Formulations of Endophytic *Bacillus* Species to Enhance Field Control of Wheat Blast

SUPPLEMENTARY

Table S1. Three-way ANOVA for culture medium optimization medium on sporulation.

Source of Variance	df	Mean square	F value	p value
Medium	2	9.787	986.50	<0.001
Bacteria	2	60.159	6064.15	<0.001
Day	2	2.279	229.69	<0.001
Medium × Bacteria	4	2.139	215.63	<0.001
Medium × Day	4	0.758	76.44	<0.001
Bacteria × Day	4	0.132	13.26	<0.001
Medium × Bacteria × Day	8	0.275	27.73	<0.001
Residual	135	0.010	-	-

Note: Statistical significance was considered at $p < 0.05$. – indicates not applicable.

Table S2. Three-way analysis of variance (ANOVA) for the effect of salt supplementation on bacterial sporulation.

Source	df	Mean square	F value	p value
Medium	4	3.342	2786.14	<0.001
Bacteria	2	77.354	64495.64	<0.001
Day	2	0.138	115.31	<0.001
Medium × Bacteria	8	0.290	241.51	<0.001
Medium × Day	8	0.011	9.55	<0.001
Bacteria × Day	4	0.059	49.15	<0.001
Medium × Bacteria × Day	16	0.015	12.76	<0.001
Residual	225	0.001	-	-

Note: Statistical significance was considered at $p < 0.05$. – indicates not applicable.

Table S3. Comparative cost for the preparation of low-cost culture media

Category	Material	Quantity Required	Price (Unit)	Price of Required Quantity
NB	Nutrient broth powder	13 g	17,000 Tk/kg	221.00 Tk
	Distilled water	To 1 L	100 Tk/L	100.00 Tk
Total				321 tk./L
	Sugarcane molasses	10–30 g	50 Tk/kg	0.50-1.50 Tk

Molasses based medium	Manganese chloride ($MnCl_2 \cdot H_2O$)	0.5 g	1,200 Tk/kg	0.60 Tk
	Sodium hydroxide (NaOH)	As required	1,700 Tk/kg	Depends on quantity used
	Distilled water	To 1 L	100 Tk./L	100.00 Tk*
Total				102-103 Tk./L

Note: Price may vary depends on company and origin of products

Table S4. Effect of formulation, bacterial strain, and storage duration on *Bacillus* viability (three-way ANOVA). Analysis was performed on log-transformed viable bacterial population (log CFU).

**Source of variation**	**df**	**Mean square**	**F value**	**p value**
Formulation	2	289.45	138781.3	<0.001
Bacterial strain	2	15.28	7325.15	<0.001
Storage duration	6	105.87	50759.21	<0.001
Formulation × strain	4	2.38	1143.49	<0.001
Formulation × storage duration	12	8.99	4309.98	<0.001
Strain × storage duration	12	0.35	168.22	<0.001
Formulation × strain × storage duration	24	0.44	213.26	<0.001

Note: Statistical significance was considered at $p < 0.05$.

Table S5. Three-way ANOVA for disease suppression and yield-related parameters of wheat under field conditions.

**Source of variation**	**Df**	**Seedling DSR (%)**	**Field DSR (%)**	**Thousand grain weight (%)**	**Relative Yield increase (%)**	**Df**	**Total grain yield plot^{-1} (g)**
Replication	2	4.12*	1.07^{NS}	7.74**	7.38**	2	0.76^{NS}
Bacteria	3	0.94^{NS}	5.32**	30.02***	47.13***	6	137.37***
Formulation	1	0.02^{NS}	2.82NS	178.31***	104.59***	1	111.49***
Method	1	8.96**	80.50***	48.34***	16.25***	1	17.50***
Bacteria × Formulation	3	0.11^{NS}	5.37**	1.45^{NS}	3.27*	3	3.45*
Bacteria × Method	3	0.30^{NS}	4.31*	0.42^{NS}	2.79^{NS}	3	2.95*
Formulation × Method	1	0.23^{NS}	2.94^{NS}	5.66*	17.41***	1	18.63***
Bact × Formulation × Method	3	0.35^{NS}	0.39^{NS}	3.21*	1.34^{NS}	3	1.42^{NS}
Residuals (30)	30					36	

Note: Significance codes: NS = not significant ($P > 0.05$); * = $P \le 0.05$; ** = $P \le 0.01$; *** = $P \le 0.001$.

Table S6. Field disease severity of wheat blast under different bacterial treatments

	Disease severity (Mean ± SE)					
Formulation × Method	BTS-3	BTS-4	BTLK6A	Consortium	Negative control	Positive control
Tlc-P	9.75 ± 0.59	9.62 ± 0.95	12.66 ± 0.40	13.92 ± 1.18	39.90 ± 1.34	4.27 ± 0.65
Tlc-F	15.83 ± 0.91	16.72 ± 0.65	14.38 ± 1.52	20.39 ± 1.66		
Mls-P	14.02 ± 1.18	10.66 ± 1.37	14.65 ± 0.73	13.37 ± 0.64		

Mls-F	18.14 ± 0.59	16.35 ± 1.56	16.06 ± 1.11	16.76 ± 1.21		

Table S7. Two-way Analysis of variance for tiller number increase (%) by seed priming with *Bacillus*-formulation.

Source of variation	Df	Sum Sq	Mean Sq	F value	Pr(>F)	Significance
Replication	2	32.91	16.46	10.52	0.0016	**
Bact	3	143.31	47.77	30.54	<0.001	***
Formulation	1	15.62	15.62	9.98	0.007	**
Bact:Formulation	3	1.7003	0.56676667	0.36	0.78119718	NS
Residuals	14	21.899425	1.56424464			-

Note: Significance codes: NS = not significant (P > 0.05); * = P ≤ 0.05; ** = P ≤ 0.01; *** = P ≤ 0.001.

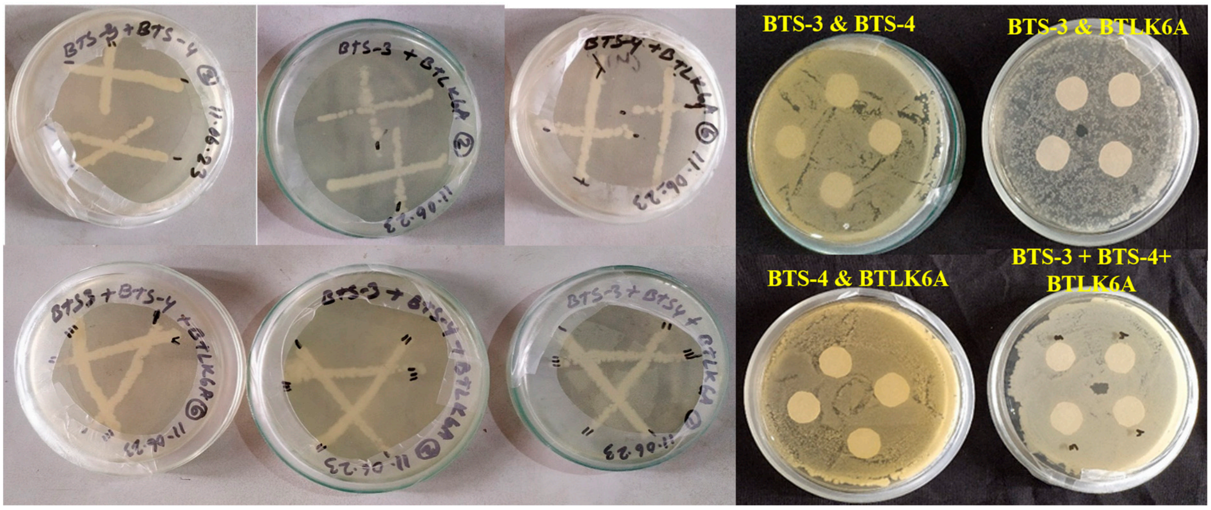

Scheme S1. Compatibility of *Bacillus* strains BTS-3, BTS-4, and BTLK6A assessed by cross-streak plate and agar-based diffusion assays.
